# Mechanisms of cell size regulation in slow-growing *Escherichia coli* cells: discriminating models beyond the adder

**DOI:** 10.1038/s41540-024-00383-z

**Published:** 2024-05-29

**Authors:** César Nieto, César Augusto Vargas-García, Juan Manuel Pedraza, Abhyudai Singh

**Affiliations:** 1 Department of Physics, Universidad de los Andes, Bogotá, Colombia; 2 Department of Electrical and Computer Engineering, University of Delaware, Newark, DE 19716 USA; 3 AGROSAVIA Corporación Colombiana de Investigación Agropecuaria, Mosquera, Colombia; 4 Department of Electrical and Computer Engineering, Biomedical Engineering, Mathematical Sciences, Center of Bioinformatic and Computational Biology, University of Delaware, Newark, DE 19716 USA

**Keywords:** Stochastic modelling, Cell biology, Cellular noise

## Abstract

Under ideal conditions, *Escherichia coli* cells divide after adding a fixed cell size, a strategy known as the *adder*. This concept applies to various microbes and is often explained as the division that occurs after a certain number of stages, associated with the accumulation of precursor proteins at a rate proportional to cell size. However, under poor media conditions, *E. coli* cells exhibit a different size regulation. They are smaller and follow a *sizer-like* division strategy where the added size is inversely proportional to the size at birth. We explore three potential causes for this deviation: degradation of the precursor protein and two models where the propensity for accumulation depends on the cell size: a nonlinear accumulation rate, and accumulation starting at a threshold size termed the *commitment size*. These models fit the mean trends but predict different distributions given the birth size. To quantify the precision of the models to explain the data, we used the Akaike information criterion and compared them to open datasets of slow-growing *E. coli* cells in different media. We found that none of the models alone can consistently explain the data. However, the degradation model better explains the division strategy when cells are larger, whereas size-related models (power-law and commitment size) account for smaller cells. Our methodology proposes a data-based method in which different mechanisms can be tested systematically.

## Introduction

To maintain tight distributions on size, bacteria must control the division time based on their size^1,2^. Recent measurements indicate that bacteria, such as *Escherichia coli* and *Bacillus subtilis*, regularly divide using the *adder* division strategy^3,4^, where the size (Δ) added during a division cycle (birth to division) is not correlated with the size at birth (*s*_*b*_)^5,6^. The molecular mechanisms behind *adder* division in bacteria are complex including the coordination of several processes, such as septal ring formation, DNA replication, and cell wall synthesis^4,7–11^. A recent hypothesis suggests that a single factor, the accumulation of the *FtsZ* protein, is the main contributor to determining the timing of division under optimal growth conditions^4^. *FtsZ* forms a ring at the future division site and recruits other proteins to build a division apparatus^12^. Mathematical models describe the accumulation of *FtsZ* as a stochastic counting process with rates that depend on the size of the cell^13–15^ opening new frontiers in the modeling of cell dynamics^16–18^. However, the exact dynamics of the *FtsZ* accumulation and the conditions under which it is the main contributor to the division remain unclear.

The *adder* mechanism can be broken under slow growth conditions^4,19^. Then, Δ is negatively correlated with *s*_*b*_. This deviation from the *adder* is known as *sizer-like* strategy^4,9,19,20^ because it is an intermediate strategy between the *adder* and the *sizer*. In this last strategy, cells divide once they reach, on average, a specified size. This transition from *adder* to *sizer-like* by changing growth conditions could reveal more information on the division process and its regulation by different factors.

The study of possible mechanisms of division, especially under slow growth conditions, has recently received increasing attention. The discovery that *FtsZ* is one important factor for division in *E. coli*^4^, has led to several studies suggesting the origins of the *sizer-like* strategy. here is evidence supporting, mainly, the degradation of *FtsZ* by the *clpX* enzyme^4,17^ and the limitation of the initiation of division by the initiation of chromosome replication^21–23^. The importance of understanding the *sizer-like* has prompted multiple groups to propose data analysis methods to distinguish between different models of division^7,24,25^, opening a debate with no clear conclusions yet. Here, we consider a general model that unifies three competing models as particular cases: (a) degradation of *FtsZ*^4,26^, which assumes that these division regulator molecules have a life span shorter than the doubling time; (b) non-linear size dependence of the *FtsZ* accumulation rate^13,19^, and (c) additional size control mechanism^9,23,27^, which posits that *FtsZ* accumulation, and thus division, only starts when the cell reaches a minimal size. This commitment between the cell cycle stages and a certain size is common in the analysis of the cell cycle regulation for different microorganisms^28,29^.

We observe that the three proposed models can fit the profile of Δ versus *s*_*b*_ by themselves^4,19^. However, it is difficult to experimentally test which mechanism better determines the origin of the *sizer-like* in each particular condition, as it requires experimental methods based on molecular biology that are not always easily accessible^4,21,23^, especially for organisms different from *E. coli*. This research will present a data-based method in which, by comparing the predictions statistically with the data and not needing the measurement of other variables such as the amount of division regulatory molecules or the instant of the initiation of chromosome replication, it is possible to estimate which model has higher probability of explain the observations.

The paper is organized as follows: we first introduce the three models of bacterial division as special cases of a general stochastic counting process. We obtain the distributions of size at division by numerically solving the corresponding forward Kolmogorov equation for each model. We evaluate the models against the existing data sets^4,19^, using not only their mean trends but also their full distributions. We apply the Akaike Information Criterion (AIC), a likelihood-based method, to measure the fit of the models penalizing their complexity. We find that none of the models can explain the *sizer-like* strategy consistently, but some models perform better than others depending on how negative the correlation Δ vs. *s*_*b*_. Finally, we discuss the implications of our findings and suggest further experiments to investigate more aspects of cell division.

## Methods

### Modeling division in rod-shaped bacteria

**Fig. 1:**
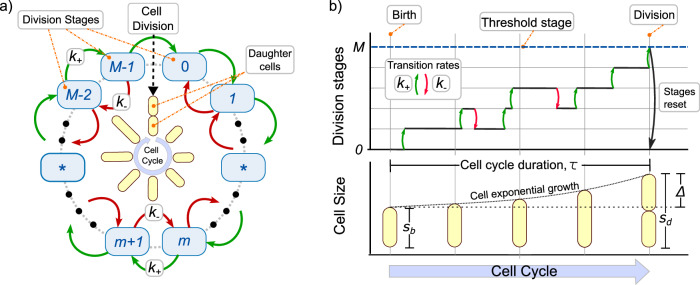
A general multistep model for triggering cell division. **a** Diagram of the cell cycle explaining how division occurs at crossing *M* stages. **b** While bacteria grow exponentially (lower panel), the division stages accumulate at rate *k*_+_ and might revert at rate *k*_−_ (upper panel). Once a number of steps *M* is reached, the cell divides, the steps are reset to zero, and the size is halved. The main variables of the bacterial division cycle are also shown: size at birth *s*_*b*_, size at division *s*_*d*_, and added size Δ = *s*_*d*_ − *s*_*b*_.

We consider the division process as the completion of a certain number of stages. During cell growth, as Fig. 1 shows, division occurs exactly when cell crosses a fixed number of stages *M*^14,30^. From a biological perspective, a possible interpretation of the division stages is related to the accumulation of a precursor protein, usually the *FtsZ* mentioned above^4,31^. However, other molecules could also be the main contributor to bacterial division^32–35^. Therefore, we use *division stages* instead of *number of precursor proteins* to explicitly keep our approach as general as possible.

Recent mathematical models have proposed cell-size based division rates^36^, multi-stages^13,19^ and back transitions^4^ to explain the division strategy. However, none of them can account for all the observed properties of cell division^24^. In this article, we present a model that encompasses each of these models as a special case and propose a likelihood-based method to test the predictions with data. We aim to provide a tool for hypothesis testing in future experiments.

A cell cycle is defined as the set of processes occurring during two consecutive divisions (Fig. 1). During the cell cycle, the cell size *s* grows exponentially over time *t* with growth rate *μ*. This means that the cell size follows:

1 $$\frac{ds}{dt}=\mu s.$$

In the *adder* strategy, the transition between stages occurs at a rate proportional to the current cell size^14,19^. To explain the *sizer-like* strategy, we will generalize the accumulation rates. The rate of stage increase *k*_+_ is considered nonhomogeneous and, depending on the model, stages can revert (by protein degradation) with a rate *k*_−_. At cell birth, that is, at the beginning of the cell cycle, the cell starts from stage *m* = 0 and size *s*_*b*_ (which can differ from cell to cell). While cells grow exponentially, stages accumulate. When reaching the *m* = *M* stages, the cell divides. Exactly before division, the cell has a size *s*_*d*_ such that the added size Δ is defined as the difference Δ = *s*_*d*_ − *s*_*b*_. Finally, during cell splitting, cell size is halved and the stages are reset to *m* = 0.

To describe stage accumulation in the cell cycle, let *P*_*m*_(*t*) be the probability that *m* ≤ *M* stages will be completed at time *t* with *t* = 0 being the beginning of the cycle. Given the rates *k*_+_ and *k*_−_, the dynamics of these probabilities are described by the master equation^37^:

2 $$\begin{array}{ll}\frac{d{P}_{0}}{dt}\,=\,-{k}_{+}{P}_{0}+{k}_{-}{P}_{1}\\ \,\qquad\vdots \,\\ \frac{d{P}_{m}}{dt}\,=\,{k}_{+}{P}_{m-1}-{k}_{+}{P}_{m}-{k}_{-}{P}_{m}+{k}_{-}{P}_{m+1}\\ \,\qquad\vdots \,\\ \frac{d{P}_{M}}{dt}\,=\,{k}_{+}{P}_{M-1}.\end{array}$$

*P*_*M*_ is the probability of reaching the target step *M* or, equivalently, the probability of the division event to occur. Since after division the cell stars at stage *m* = 0, the initial condition (*t* = 0) is considered as *P*_*m*_(*t* = 0) = *δ*_*m*,0_ with *δ*_*i*,*j*_ being the Kronecker delta function.

**Fig. 2:**
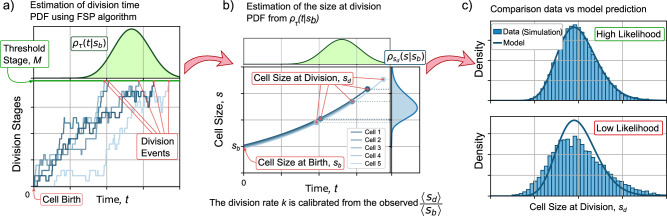
Process to predict the distribution of size at division and its comparison with data. **a** The distribution of division times *τ* given the size at birth *s*_*b*_ is estimated by solving numerically the master equation (2). **b** The size distribution at division *s*_*d*_ is obtained from the distribution of division times and considering the exponential growth using (7). **c** The comparison with the data is made using methods based on likelihood. The distribution with higher likelihood fits the data better than a distribution with lower likelihood.

As shown in Fig. 2a, we are interested in the estimate of the time to division *τ*. *P*_*M*_ is related to *τ* as:

3 $${P}_{M}(t):= {\mathbb{P}}\{\tau \in (0,t)\}.$$

This is, *P*_*M*_(*t*) is also the probability that *τ* occurs in the interval (0, *t*). Hence, the probability density function PDF (also known as the distribution) of the division time *ρ*_*τ*_ is related to *P*_*M*_ following:

4 $${P}_{M}(t)=\int\nolimits_{0}^{t}{\rho }_{\tau }({t}^{{\prime} })d{t}^{{\prime} }.$$

After estimating *P*_*M*_ by solving (2), *ρ*_*τ*_(*t*) can be calculated as follows^38^:

5 $${\rho }_{\tau }(t)=\frac{d{P}_{M}}{dt}={k}_{+}{P}_{M-1}.$$

As shown in Fig. 2b, the PDF of the cell size at division *s*_*d*_ can be estimated from *ρ*_*τ*_, considering that the cells grow exponentially. This is, the cell size *s* is related to the size at birth *s*_*b*_ through the time from birth *t*:

6 $$s={s}_{b}{e}^{\mu t};\quad t(s)=\left(\frac{1}{\mu }\right)\ln \left(\frac{s}{{s}_{b}}\right).$$

A transformation of variables allows us to obtain the PDF of sizes at division ${\rho }_{{s}_{d}}(s)$ as:

7 $${\rho }_{{s}_{d}}(s)={\rho }_{\tau }(t(s))\frac{dt}{ds},$$

where $\frac{dt}{ds}=\frac{1}{\mu s}$ when exponential growth (1) is assumed. Observe how, since the cell size depends on *s*_*b*_, these distributions also depend on *s*_*b*_.

As explained in Fig. 2b, a comparison between experiment and theory requires the calibration of the model parameters. This is done by estimating the moments of *s*_*d*_ using the estimated ${\rho }_{{s}_{d}}(s)$ in (7). If 〈. 〉 defines the averaging operator, the *α*-moment of the distribution of *s*_*d*_, written as $\langle {s}_{d}^{\alpha }\rangle$, is defined as follows,

8 $$\left\langle {s}_{d}^{\alpha }\right\rangle =\int\nolimits_{{s}_{b}}^{\infty }{s}^{\alpha }{\rho }_{{s}_{d}}(s)ds,$$

where the mean size at division 〈*s*_*d*_〉, the moment with *α* = 1, can be used to calculate the mean added size per division cycle 〈Δ〉 = 〈*s*_*d*_〉 − *s*_*b*_ as a function of the size at birth *s*_*b*_. The particular parameters of the model (as will be explained later) are adjusted, such as the predicted 〈*s*_*d*_〉 is constrained to the observed average 〈*s*_*d*_〉 assuming that the mean size at birth 〈*s*_*b*_〉 = 1.

After imposing this constraint, the best model parameters are adjusted to the data by maximizing the likelihood function. As shown in Fig. 2c, the likelihood function measures the precision of the PDF with the histogram associated with the data. With a higher likelihood, the predicted distribution fits the experiment better.

The moments of *s*_*d*_ can also be used to quantify the noise in added size $C{V}_{\Delta }^{2}$, the ratio between var(Δ) (the variance of Δ) and 〈Δ〉^2^, which is a measure of the stochastic variability of Δ. We can obtain $C{V}_{\Delta }^{2}$ as a function of *s*_*b*_ from ${\rho }_{{s}_{d}}$ and its moments $\langle {s}_{d}^{2}\rangle$ and 〈*s*_*d*_〉 using the formula^19^:

9 $$C{V}_{\Delta }^{2}=\frac{{{{\rm{var}}}}(\Delta )}{{\langle \Delta \rangle }^{2}}=\frac{\langle {s}_{d}^{2}\rangle -{\langle {s}_{d}\rangle }^{2}}{{(\langle {s}_{d}\rangle -{s}_{b})}^{2}}.$$

Observe how since $\langle {s}_{d}^{\alpha }\rangle$ depends on *s*_*b*_, different models may predict different trends on $C{V}_{\Delta }^{2}$. For us, while the trend 〈Δ〉 vs *s*_*b*_ is known as the division strategy, $C{V}_{\Delta }^{2}$ vs *s*_*b*_ is the noise signature of the model. Next, we will explain these models as particular cases of *k*_+_ and *k*_−_.

### The *Adder* strategy

The implementation of the *adder*, where 〈Δ〉 is independent on *s*_*b*_ corresponds to the particular case of (2) where *k*_+_ and *k*_−_ are given by^19^:

10 $${k}_{+}=ks;\quad {k}_{-}=0,$$

with *k* a constant and *s* = *s*_*b*_*e*^*μt*^ is the cell size. Assuming exponentially growing cells and a division process defined by both (2) and (10), the mean added cell size 〈Δ〉 is given by^19^:

11 $$\langle \Delta \rangle =M\frac{\mu }{k},$$

which is, as expected, independent of *s*_*b*_. The noise in Δ, defined in (9) follows:

12 $$C{V}_{\Delta }^{2}=\frac{1}{M},$$

which is also uncorrelated with *s*_*b*_ as observations suggest^19^.

### *Sizer-like* by molecule degradation

As^4^ suggests, *sizer-like* division strategy can be obtained by including active degradation of the division-triggering molecules. In our framework, degradation is equivalent to a step backward in the accumulation of *M* stages. In this case, the rates *k*_+_ and *k*_−_ are given by:

13 $${k}_{+}=ks;\quad {k}_{-}=\gamma m,$$

where *γ* is the rate at which each molecule is degraded.

**Fig. 3:**
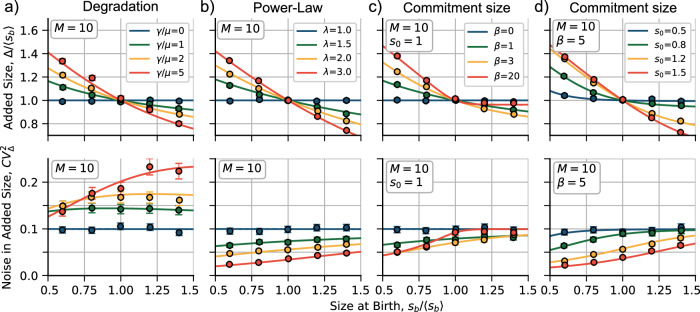
Trends on mean added size before division 〈Δ〉 (top) and its stochastic fluctuations (noise) $C{V}_{\Delta }^{2}$ (bottom) as functions of the size at birth *s*_*b*_. **a** Trends considering the model of degradation for different values of the degradation rate *γ* relative to the growth rate *μ* in (13). **b** Predictions considering a division rate proportional to a power of size for different exponents *λ* in (14). **c**, **d** Predictions considering a commitment size with different values of the Hill function exponent *β* and the commitment size *s*_0_, respectively, in (15). The trend lines correspond to the numerical solutions of (2). Large dots are obtained from simulations. Error bars represent the 95%-confidence interval over 10000 simulated cycles. The constant *k* is set in each case such as 〈Δ〉 = 〈*s*_*b*_〉 = 1. Other parameters are shown inset.

A negative slope is obtained in Δ vs *s*_*b*_ as shown in Fig. 3a (top). The higher *γ*, the more pronounced the slope. This model reduces to the *adder* when *γ* ≪ *μ*. The noise signature of this model is presented in Fig. 3a (bottom) where, as the main property, we can see that for an increase *γ*, for fixed division steps *M*, it is expected a higher average $C{V}_{\Delta }^{2}$.

### *Sizer-like* via non-linear division rate

Following^19^, we consider a scenario where a molecule that triggers division is produced at a rate that depends on a power *λ* of cell size *s*, this is:

14 $${k}_{+}=k{s}^{\lambda };\quad {k}_{-}=0.$$

After substituting (14) in (2), plus the assumption of exponential growth, the division strategy exhibits a *sizer-like* behavior when *λ* > 1 (Fig. 3b top). For the particular case of *λ* = 1, the model reduces to the *adder*. Hence, the higher *λ*, the higher the slope of the relationship between Δ and *s*_*b*_. The power law on the production rate also affects the fluctuations of the added size $C{V}_{\Delta }^{2}$: it lowers the average noise level for a given *M* and simultaneously increases the positive slope of $C{V}_{\Delta }^{2}$ versus *s*_*b*_ (Fig. 3b bottom).

### *Sizer-like* via a commitment size

Recent evidence suggests that cells may aim for a minimal size before starting division programs^9^. We will denote this minimal or commitment size by *s*_0_. We propose that it can be incorporated into our framework as

15 $${k}_{+}=\frac{ks}{1+{({s}_{0}/s)}^{\beta }};\quad {k}_{-}=0.$$

By replacing (15) with (2), we can see that the division strategy has a *sizer-like* behavior when *β* > 0 (Fig. 3c top). The parameter *β* can control the slope of the curve Δ versus *s*_*b*_ for a fixed *s*_0_. It also influences the fluctuations of the added size $C{V}_{\Delta }^{2}$: It reduces the average noise level for a given *M*, but increases the positive slope of $C{V}_{\Delta }^{2}$ versus *s*_*b*_ (Fig. 3c bottom). This model encompasses the *adder* strategy as a special case when *β* = 0.

The commitment size *s*_0_ relative to the mean size at birth 〈*s*_*b*_〉 also affects the division strategy. For a fixed *β*, a low *s*_0_ ≪ *s*_*b*_ mimics the *adder*, while a high *s*_0_ ≫ *s*_*b*_ approximates the *sizer* (the strategy where the slope in Δ versus *s*_*b*_ is −1). This is because cells born with a size below *s*_0_ follow a perfect *sizer* strategy, while cells born with size above *s*_0_ follow the *adder*. For small *β*, the *adder* strategy is recovered. For intermediate *β*, the transition from *sizer* to *adder* is smoother as *s*_*b*_ increases.

### Comparison: theory versus datasets

We have shown how to derive the PDF *ρ*(*s*_*d*_∣*s*_*b*_) from different models. Now, we want to estimate how accurate these distributions are relative to the data. We use the Akaike Information Criterion (AIC)^39^ to reward the fit of the models to the data while penalizing the number of free parameters. The adder model has one parameter (*M*), the degradation and power-law models have two (*γ*, *M* and *λ*, *M* respectively), and the commitment size model has three (*s*_0_, *β* and *M*). Taking into account all experiments, for each pair of data (*s*_*b*_, Δ) normalized by 〈*s*_*b*_〉 we numerically compute the size-at-division distribution ${\rho }_{{s}_{d}}(s| {s}_{b})$ given *s*_*b*_ using (7). We find the parameters that maximize the likelihood function^40^ based on the data. Then we calculate the AIC for each model. The model with the lowest AIC is the most probable one, and we denote its AIC value by *AIC*_*min*_. To compare the relative probability of each model with the most probable one, we use the concept of relative likelihood^41^. For a model *i* with an AIC value of *AIC*_*i*_, the relative likelihood *p* is given by:

16 $$p=\exp [(AI{C}_{min}-AI{C}_{i})/2].$$

**Fig. 4:**
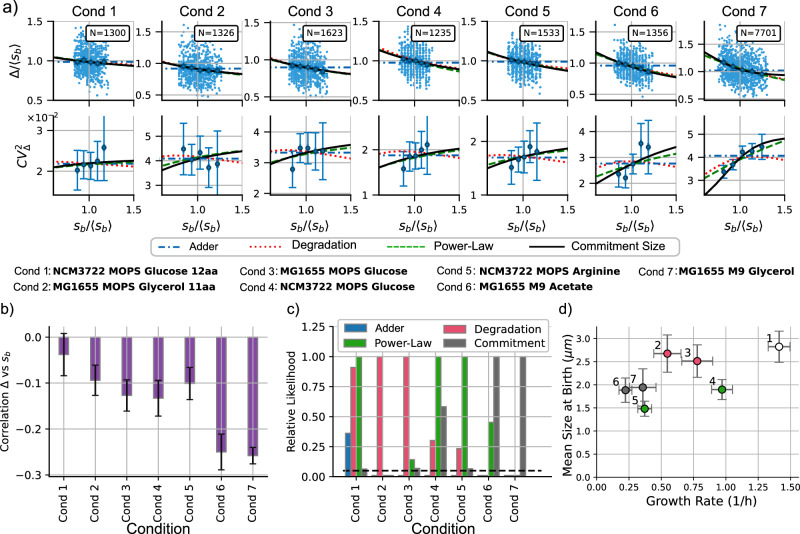
Discriminating between models across different experimental conditions. **a** Top: trends of the added size Δ vs the size at birth *s*_*b*_. Bottom: Noise signatures, quantified by stochastic fluctuations of the added size $C{V}_{\Delta }^{2}$ versus *s*_*b*_. The numerical prediction of the three models can be better discriminated: the degradation model (red dotted line), the power law (green dashed line), and the commitment size (black solid line). *N* represents the number of studied cell cycles. *Cond* represents the condition number. In the middle of the figure, more detailed labels are shown. Error bars represent the 95% CI using bootstrapping methods. **b** Comparison of the correlation function between Δ and *s*_*b*_ for different conditions. The more negative this correlation is, the closer to *sizer* the division strategy is. **c** Relative likelihood of each model with respect to the model with the best AIC score using (16). The black dashed line represents the relative likelihood of 0.05. **d** Different conditions discriminated by the mean cell size at birth versus the mean growth rate. The color of the dots represents the most probable model, and the error bars represent the standard deviation of each variable.

The AIC method is useful for estimating the accuracy of the models but is not easy to visualize since the match of the distributions is very similar. To gain a better intuitive understanding of how each model behaves, we can use the method of statistical moments. As shown in Fig. 3, this method involves plotting the division strategy (〈Δ〉 versus 〈*s*_*b*_〉) and the noise signature ($C{V}_{\Delta }^{2}$ versus 〈*s*_*b*_〉) and comparing them with the data. From theory, we can obtain the moments directly: given a *s*_*b*_, they are calculated from the distribution using (8). From the data set, we visualize the moments given *s*_*b*_ using quantile splitting. This method splits the data into a given number of quantiles and computes the statistics for each quantile separately. The points in Fig. 3 (five quantiles) represent the data from simulations, while Fig. 4a (also five quantiles) represents the experimental data. To study the division strategy, we plot ${\langle {s}_{b}\rangle }_{q}$ and 〈Δ〉_*q*_ for each quantile. The noise signature is obtained by plotting the variance ${\langle (\Delta -{\langle \Delta \rangle }_{q})\rangle }^{2}/{\langle \Delta \rangle }_{q}^{2}$ for each quantile. Error bars indicate a 95% confidence interval using bootstrapping methods.

### Experiments

We analyzed two independent already published datasets of *E. coli* strains under different growth conditions^4,19^. Data were obtained from time-lapse microscopy images of confined single cells fed in a *mother machine* micro-fluidic device. References^4,19^ imaged slow-growing cells corresponding to steady growth conditions.

We measured the cell size using the cell length since the width is approximately constant. We normalize all lengths by the mean size at birth 〈*s*_*b*_〉. The theoretical division rate *k* was estimated, given the free parameters and setting the growth rate at $\mu =\ln (2)$, from the observed 〈Δ〉 with 〈*s*_*b*_〉 = 1. Besides simplify our computations, with this time and cell-size normalization, our idea is to obtain general results. Our conclusions should be scale-free because we only study dimensionless quantities such as the slope and correlation between Δ vs. *s*_*b*_ and noise in added size $C{V}_{\Delta }^{2}$. These properties of cell division emerge naturally given the model parameters. The experimental data and the data analysis scripts are available at^42^.

## Results

Figure 4a shows the comparison between the data and the theory. First, we study the case where the division strategy is close to *adder*, that is, when Δ is independent of *s*_*b*_. This occurs for condition 1 (NCM3722 in MOPS with Glucose) since the 95% confidence interval for the correlation includes zero (Fig. 4b). For these conditions, the adder model has a relatively high AIC, but not as high as the other three models. The three models reach a similar likelihood, but the commitment size model is punished for its complexity. Both degradation and power-law models reach similar AIC scores, and it is not possible to discard one of those models with enough confidence.

For the other conditions, the division is *sizer-like* with statistical significance (Fig. 4c). In Fig. 4c, we observe that the degradation model has a minimum AIC (and therefore highest relative likelihood) for cells with a larger mean size at birth (conditions 2 and 3, MG1655 in MOPS with glycerol 11aa and MOPS with Glucose). For smaller strains, power-law and commitment show lower AICs. Power-law has the lowest AIC for conditions 4 and 5 (NCM3722 in MOPS glucose and MOPS arginine), and the commitment size model performs best for cell conditions with the most negative correlation Δ vs *s*_*b*_ (conditions 6 and 7: MG1655 in M9 with Acetate and M9 with Glycerol).

To better visualize the performance of the models, we can compare the statistical moments with the binned moments of the data. In Fig. 4a, we observe that all of our proposed models capture the mean trend in Δ vs *s*_*b*_. However, they differ in noise signature ($C{V}_{\Delta }^{2}$ vs *s*_*b*_). The degradation model predicts a low correlation between $C{V}_{\Delta }^{2}$ and *s*_*b*_, while the power-law model predicts an increasing function. The commitment size model behaves like the power law when the slope of Δ vs *s*_*b*_ is small but predicts a higher slope of $C{V}_{\Delta }^{2}$ vs *s*_*b*_ when the slope of Δ vs *s*_*b*_ is large.

## Discussion

In this paper, we propose a general model that includes different mechanisms of cell division regulation with experimental evidence. Each of them is a particular case. We found that each mechanism contributes differently to division depending on the growth condition. Our method can be used as a tool for the study of the origin of different division strategies not only in *E. coli* but also in other microorganisms. It includes complex observable variables such as the growth rate or the division-initiation size. We believe that our model can be generalized to other scenarios, such as cycle-stage-dependent growth rate or size-independent division transition rates, as seen in eukaryotic cells.

The transition from the *adder* to the *sizer-like* division strategies suggests the presence of a dual mechanism governing cell division: a primary mechanism that leads to the *adder* being the most important for larger cells, and a secondary mechanism that gives rise to the *sizer-like* is more visible in smaller cells. It is plausible that this secondary mechanism has often been overlooked in laboratory settings, where cells are commonly cultivated in nutrient-rich environments and are relatively large. However, this mechanism is of substantial importance, as it could elucidate cell survival and adaptation in real-world scenarios characterized by less-than-optimal or slower growth conditions.

Si et al.^4^ found experimental evidence that *FtsZ* degradation could be the basis for the *sizer-like* strategy. After inhibiting the production of ClpXP, an ATP-dependent protease^43^ that degrades FtsZ, *E. coli* cells that showed a *sizer-like* restored the *adder* division. Our investigation highlights the potency of the degradation model in explaining the *sizer-like* strategy, particularly when the relationship between added size and birth size is not excessively steep. However, for steeper slopes, alternative mechanisms align better with the data. We believe that the actual mechanism that explains *sizer-like* is a composition of degradation and commitment size since they are not exclusive.

The commitment size model posits a minimum size prerequisite for division initiation, which yields the *sizer-like* behavior. When the cell size at birth is relatively small, it often falls below this commitment size, needing to grow until reaching this threshold. Then the division machinery starts to build up. In contrast, when cells at birth exceed the commitment size, division starts immediately. With division starting just after birth, we expect large cells to follow the *adder* strategy. A visual representation of how the cell size plays a role in defining which model is more important is presented in Fig. 4d, where cells with a mean size at birth smaller than 2.3 μm (conditions 4–7) have a higher probability of being explained by a size-dependent division rate (either power law or commitment size models). Cells with a larger size (conditions 2 and 3) at birth are more likely to have a *sizer-like* by molecule degradation. Condition 1 has almost no correlation Δ versus *s*_*b*_ and does not have a clear main contributor. In contrast, the average growth rate does not appear to define the main contributor to the division strategy.

This work shows that different models can fit the mean behavior, but the noise signature can be used to distinguish them. However, we acknowledge some limitations of these fluctuations-based methods. The added size distribution has multiple noise sources in addition to randomness related to the control mechanism; we can include others, such as instrumental precision: low camera resolutions, segmentation errors, and length as a size proxy that ignores fluctuations in cell width^18^. These uncertainties can add noise to the added size, but we believe that they cannot explain the high level of noise we observe and that the noise component of the division mechanism is high enough to neglect these instrumental sources.

The power-law rate model operates on the premise that the division rate has a stronger dependence on the size than the *adder* model. It is important to note that this model is heuristic, using nonlinear size dependency as an effective parameter to capture the intricacies of division. Thus, the power-law rate may approximate the Hill function rate of the commitment size model within a specific range. In contrast, the commitment size model, with more free parameters, can exhibit greater adaptability compared to the power-law model.

The notion of commitment size might relate to the initiation of chromosome replication at a fixed size per origin^9,44,45^. Although our model does not explicitly integrate chromosome replication, potential links between these variables remain open. Recent studies hint at coordination between division and replication in bacteria, resembling observations in more complex organisms^35,46–48^. Other variables that we did not study can contribute to the origin of the *sizer-like*. Recently, there is evidence about the dependence of growth rate and cell size at birth^25,49^, the correlation between lineage-related cells^50^ and randomness in growth rate^51^. We think that the addition of new free parameters makes the model more complex, and our fitting metric (the AIC score) punishes the complexity. Our aim is not to over-fit the model to each condition but to find the more general conclusion with the least number of assumptions.

The underlying biological mechanism enabling the cell to measure commitment size remains a subject of active debate. One perspective suggests tight synchronization between division and DNA replication, which triggers division when reaching a target size for replication initiation^9,21,52^. Another angle posits the existence of molecules acting as size proxies within cells, regulating division initiation^24,53^. Further experiments, as proposed in^54,55^, could provide additional information on the division strategy. Dynamic environments, where the division strategy changes dynamically, could shed light on these mechanisms. Other ways to achieve slow-growth conditions can also help us understand the validity of the models. These conditions can include decreasing growth temperature and growing in the presence of a mild concentration of antibiotics. Furthermore, exploring the division strategy with the *clpX* knockdown strain under different growth conditions, coupled with the use of AIC or other likelihood-based methods for data analysis, has the potential to provide a more comprehensive understanding of these phenomena.

### Reporting summary

Further information on research design is available in the Nature Research Reporting Summary linked to this article.

### Supplementary information

Reporting summary

## Data Availability

Dataset and code used for the data analysis discussed in this manuscript were gathered in a Zenodo repository with an MIT license at the following link: 10.5281/zenodo.3951080.
